# Cross-validation of the safe supplement screener (S3) predicting consistent third-party-tested nutritional supplement use in NCAA Division I athletes

**DOI:** 10.3389/fnut.2024.1519544

**Published:** 2025-01-15

**Authors:** Kinta D. Schott, Avaani Bhalla, Emma Armstrong, Ryan G. N. Seltzer, Floris C. Wardenaar

**Affiliations:** College of Health Solutions, Arizona State University, Phoenix, AZ, United States

**Keywords:** batch testing, dietary supplements, sport foods, ergogenic aids, doping

## Abstract

**Introduction:**

This cross-sectional study aimed to cross-validate an earlier developed algorithm-based screener and explore additional potential predictors for whether athletes will use third-party-tested (TPT) supplements.

**Methods:**

To justify the initial model behind the supplement safety screener (S3) algorithm which predicts whether athletes will use TPT supplements, a cross-validation was performed using this independent dataset based on responses of a large group of collegiate NCAA DI athletes. Additionally, explorative modeling using stepwise logistic regression was used to identify new predictors for TPT supplement use to create and evaluate a new model for future use.

**Results:**

The S3 algorithm was applied to the responses from *n* = 662 athletes using supplements (age: 20 ± 1.5 years, 50% female, from >24 sports) confirming that the algorithm identifies consistent and inconsistent TPT users (χ^2^ (1) = 15.95, *p* < 0.001), with a moderate area under the curve (AUC, 0.67) and a moderate specificity (68%), but low sensitivity (51%). Explorative modeling identified two new variables: TPT logo recognition, and having at least one name, image, likeness (NIL) deal that may help to predict TPT supplement use.

**Discussion:**

Strong relationships between risk groupings and product use outcomes toward TPT supplement use were identified for the athletes screened. The S3 screener showed high sensitivity for identifying student-athletes inconsistently using TPT supplements, but a low specificity, lacking the ability to place less risky athletes into low-risk quadrants. The exploratory modeling, identifying TPT logo recognition and having a NIL deal, further strengthens our knowledge on predictors for consistent TPT supplement use.

## 1 Introduction

Athletes commonly use nutritional supplements, such as dietary supplements, sports foods, and ergogenic supplements, and most athletes use multiple products throughout the year (1). As many as 97% of athletes report the use of dietary supplements during their career for various reasons, including to promote recovery, improve performance, or improve general health (2, 3). At the same time, athletes need to adhere to regulations and guidelines of their competitive sport associations, such as the International Olympic Committee and the National Collegiate Athletics Association (NCAA). In addition, athletes need to adhere to doping regulations that their sports organizations adhere to. During competition and training, most athletes (collegiate, national and international) must follow restrictions listed by the World Anti-Doping Agency (WADA); however, in the United States, the NCAA has its own set of regulations that mostly follow WADA regulations with minor discrepancies (4). WADA aims to protect athletes from physical harm caused by substance use and to create an equal playing field by ensuring that no athlete has an unfair advantage (5).

Although food products in the United States are heavily regulated by agencies, such as the Food and Drug Administration (FDA), dietary supplements do not fall into a category which requires extensive testing for purity and efficacy prior to sale (2). Because most common dietary supplements contain ingredients that are generally recognized as safe, they do not require pre-approval from the FDA, but cannot be marketed as a treatment or cure for disease (6). A concern that arises from the lack of quality assurance on behalf of the FDA is that nearly 28% of dietary supplements pose a risk of unintentional doping and as many as 9% of reported doping cases arise from off-label ingredients (7, 8). Organizations have formed with the goal of reducing the occurrence of adulterated products and are referred to as third-party-testing (TPT) organizations. These organizations provide an external testing service to supplement manufacturers to certify the product for label verification and possible common contaminants (e.g., microbes, metals and mold), and some organizations also identify supplement adulteration from substances banned for athletes (9). Although organizations exist that focus on serving athletes, only a limited number of supplements on the market are third-party tested, and as a result adulteration is always an active risk. Additionally, these organizations do not test the product’s efficacy and do not indicate that the product of note is more beneficial than another. Based on NCAA and WADA regulations, athletes participating in competition are liable for all ingredients included in a product on- and off-label and assume responsibility for any substance consumed (7, 10). While the use of TPT nutritional supplements does not provide infallible protection for the athlete from unintentional doping, it is the consensus that athletes use TPT nutritional supplements to mitigate the risk (9, 11, 12). Although it is consistently recommended that athletes use tested products, compliance is low (14). Recently, only 38% of athletes reported the consistent use of TPT products, lower than the previously reported 57% consistency (2, 14). As a result of this inconsistency, our research group created a safe supplement screener (S3), and associated algorithm aimed at identifying risky behaviors related to inconsistent use of TPT products in a NCAA DI student-athlete population (15).

The previously created S3 algorithm from our lab (15), identified ten predictor variables both positively and negatively associated with TPT supplement use consistency. The predictors are as follows: familiarity with WADA banned substances, knowing where to find and order TPT supplements, searching for information, discussing supplement choices with a registered dietitian (RD), purchasing supplements outside of what is provided by an athletic department, taking supplement advice from friends or family, and using multivitamins, weight gainer, caffeine or creatine. The initial validation of the S3 algorithm resulted in a reasonably accurate outcome for predicting high-risk inconsistent TPT supplement users, but the algorithm lacked the specificity to classify low-risk consistent users accurately (15). The aim of the present study is to perform a multi-level cross-validation of the S3 algorithm, predicting whether elite collegiate athletes use TPT supplements in an independent dataset, confirming the 60% cut-off for the algorithm predicting a low versus high risk of not using TPT, as well as confirming a reasonable area under the curve (AUC), using newly obtained cross-validation data. Additionally, the current dataset was used to further explore other predictors related to TPT supplement use for future use in the guidance and counseling of athletes.

## 2 Materials and methods

### 2.1 Study design

The present study consisted of a cross-sectional design that questioned athletes from eight NCAA DI athletic departments between September 2023 and January 2024. The survey questioned athletes about their behaviors and attitudes involving safe nutritional supplement use. Athletes were recruited via email, in person at fuel stations, and during team meetings. The results were analyzed aiming to validate the algorithm and its included predictors for TPT supplement use as previously identified (15).

### 2.2 Study participants

Athletes participating in the present study were sampled from eight selected NCAA DI athletic departments from a single conference. The departments featured in this study were all members of the Pac-12 [i.e., the pacific-12 conference, a collegiate athletic conference in the Western United States that competes in the National Collegiate Athletic Association (NCAA) Division I]. Athletes participating at this level have been regarded as elite or international level competitors (16). Participants needed to be between 18 and 35 years of age, as well as a current member of a varsity sport at one of the participating athletic departments. Responses were excluded from the dataset if the participant fell outside the age range (18–35 years), less than 70% of the questionnaire was completed, the response was a duplicate (the first response, identified via timestamp, was saved) or the response was suspected to be bot generated (IP address was missing from Qualtrics file). A total of 725 responses were recorded after all exclusion criteria were applied.

The study was approved by the Arizona State University Institutional Review Board (STUDY00015034). Student-athletes were instructed to read informed consent and required to select a consent validation before accessing the questionnaire. The anonymous questionnaire was accessible as a link or QR-code. Once completed, student-athletes were directly linked to a separate questionnaire where personal information was requested in order to receive a $50.00 virtual gift card for completion of the questionnaire. Personal identifying information was used only as a method for sending incentives and any identifying information, including IP addresses, were removed after data analysis.

### 2.3 Questionnaire

The web-based questionnaire was administered through Qualtrics (SAP, Seattle, WA, USA), and each question required a response to move forward. The questionnaire consisted of 75 questions and was partly adapted from published literature (15) with additional Theory of Planned Behavior (TPB) questions (17–19) and newly formulated “original” questions. The questionnaire used in the present study consisted of seven main categories: Demographics (#10 Q), Information Sources (#6 Q), Social Media (#5 Q), Supplement Knowledge (#9 Q), Nutritional Supplement Use (#11 Q), Attitudes and Barriers (#13 Q), and a series of questions utilizing the Theory of Planned Behavior (#19 Q). One additional question at the beginning indicated the participant’s consent prior to completing the survey, and one question at the end asked for a single use code to allow the research team to incentivize participants via a separate questionnaire to ensure anonymity. In comparison to the original questionnaire used to develop the S3 algorithm (15), 33 new questions were developed or modified to clarify some aspects (Supplementary File 1, general questionnaire); however, all original questions included in the S3 algorithm to predict TPT supplement use were kept the same (15). For the current article, only sample’s basic demographic data (Table 1), the variables being part of the S3 algorithm (15) as mentioned in Table 2 (Supplementary File 2, S3 questionnaire), as well as the newly identified variables based on the explorative modeling in section 3.4 have been reported

**Table 1:** Demographics of the NCAA Division I collegiate athletes surveyed reported as frequency (% and n) or as mean ± standard deviation, for total athletes surveyed (*n* = 725).

	Number or percentage
Sex	
Female	50%, (*n* = 362)
Male	50%, (*n* = 363)
Age	
Years	20 ± 1.5
Sport (*n* = 740)	
Artistic Swimming (W)^*^	0%, (*n* = 1)
Baseball (M)	7%, (*n* = 51)
Basketball (M)	2%, (*n* = 11)
Basketball (W)	1%, (*n* = 4)
Beach Volleyball (W)	2%, (*n* = 15)
Cheerleading (M)	1%, (*n* = 6)
Cheerleading (W)	1%, (*n* = 5)
Cross Country (M)	2%, (*n* = 13)
Cross Country (W)	2%, (*n* = 13)
Field Hockey (W)	1%, (*n* = 7)
Football (M)	14%, (*n* = 104)
Golf (M)	1%, (*n* = 7)
Golf (W)	2%, (*n* = 12)
Gymnastics (M)	0%, (*n* = 2)
Gymnastics (W)	2%, (*n* = 17)
Ice Hockey (M)	0%, (*n* = 1)
Lacrosse (M)	3%, (*n* = 21)
Lacrosse (W)	3%, (*n* = 18)
Rowing (M)	5%, (*n* = 36)
Rowing (W)	8%, (*n* = 58)
Rowing – lightweight (W)	0%, (*n* = 1)
Rugby (M)	0%, (*n* = 1)
Skiing (M)	1%, (*n* = 6)
Skiing (W)	1%, (*n* = 9)
Soccer (M)	3%, (*n* = 21)
Soccer (W)	7%, (*n* = 52)
Softball (W)	3%, (*n* = 20)
Swimming & Diving (M)	3%, (*n* = 22)
Swimming & Diving (W)	3%, (*n* = 42)
Tennis (M)	1%, (*n* = 6)
Tennis (W)	2%, (*n* = 12)
Track and Field (M)	7%, (*n* = 54)
Track and Field (W)	7%, (*n* = 53)
Triathlon (W)	0%, (*n* = 1)
Volleyball (M)	0%, (*n* = 2)
Volleyball (W)	1%, (*n* = 10)
Water Polo (W)	1%, (*n* = 9)
Wrestling (M)	0%, (*n* = 1)
Athlete type (student-athletes could select multiple options)	
Carded Athlete	2%, (*n* = 18)
Part of national doping testing pool	3%, (*n* = 24)
Member of national team or selection	8%, (*n* = 61)
Student-athlete at a US collegiate AD	93%, (*n* = 689)
Student-athlete not at a US collegiate AD	1%, (*n* = 7)
Professional athlete	3%, (*n* = 21)
Have one or more Nam, Image, Likeness (NIL) deals	14%, (*n* = 104)
Have received nutrition information, counseling, or advice during the last 12 months from any of the people or professions^‡^	
Sports RD within AD	84%, (*n* = 621)
Sports RD outside of AD	9%, (*n* = 67)
I have not received nutrition information	12%, (*n* = 92)
Number of visits completed for nutrition information, counseling, or advice during the last 12 months^‡^^‡^	
*Sports RD within AD (n = 586)*	
1–2 visits	48%, (*n* = 282)
3–6 visits	36%, (*n* = 211)
7–10 visits	8%, (*n* = 48)
11 or more visits	8%, (*n* = 45)
*Sports RD outside of AD (n = 60)*	
1–2 visits	63%, (*n* = 38)
3–6 visits	28%, (*n* = 17)
7–10 visits	5%, (*n* = 3)
11 or more visits	3%, (*n* = 2)
Dietary supplement use (sport foods with a nutrition facts label not included)	
Non-users	9%, (*n* = 63)
At least one self-reported supplement	91%, (*n* = 662)

* One response could not be placed in a category as the athlete indicated to be male while selecting synchronized swimming, a sport with no males on the rosters.

** Acronyms: Men’s sport (M), Women’s sport (W), Athletic department (AD), Registered Dietitian (RD).

^‡ Percentages may not add to 100% as student-athletes could select multiple options.^

^‡‡ Samples based on previous question response, student-athletes could select only one option.^

**Table 2:** Output of cross-validation for variables identified by previous algorithm-based screener (15) as predictors for athletes classified as a consistent vs. inconsistent or a non-user of TPT nutritional supplements (excluding sports foods) using *n* = 662 NCSS DI collegiate athletes.

Variable	Prevalence (% yes)	DF	Estimate	Standard error	Wald Chi-Square	Pr > Chi-Square
Intercept	–	1	0.54	0.36	2.23	0.136
**WADA Familiarity**: Are you familiar with banned substances that may occur in nutritional supplements listed on the WADA (world anti-doping agency) list? (Select only one – yes/no)	54%	1	−0.49	0.28	3.02	**0.082**
**Knowing where to find & order TPT supplements**: I know where to find and order third-party tested supplements. (Only select one – yes/no)	53%	1	−0.64	0.30	4.57	**0.033**
**Search for information**: Where do you go to look for information on nutritional supplements and sports foods? (Check all that apply – checked score: I do not search for information on my own)	22%	1	0.81	0.36	5.15	**0.023**
**Discussing supplement choices with RD**: I discuss all my supplement choices with the Athletic Departmental Sports RD. (Only select one – yes/no)	62%	1	−0.81	0.28	8.24	**0.004**
**Purchase outside athletic department**: Do you purchase or use nutritional supplements outside what is provided by your Athletic Department? (Only select one – yes/no)	45%	1	0.53	0.29	3.33	**0.068**
**Advice from others**: I’ve decided to purchase one or more supplements as a result of the advice of family, friends, or teammates. (Only select one – yes/no)	43%	1	0.57	0.28	4.19	**0.041**
Please check all of the following nutritional supplements you have used during the last 12 months. (Check all that apply)						
**Multivitamin**: (Multivitamin and mineral supplement checked)	51%	1	−0.44	0.27	2.53	0.112
**Weight gainer**: (Weight gainer checked)	5%	1	−0.96	0.56	2.89	**0.089**
**Caffeine**: (Caffeine checked)	54%	1	1.34	0.28	22.89	**<0.0001**
**Creatine**: (Creatine checked)	30%	1	−0.62	0.33	3.55	**0.059**

These predictors were previously identified by Wardenaar et al. (19). DF = Degrees of freedom; Estimate = the weight and direction of the variable within the model; Wald Chi-Square = Test to determine if independent variables are significant for the model; Pr > Chi-Square = *p* – value that preferable was below or close to 0.10 as determined before the start of the modeling process. Note: bolded values indicate statistical significance.

### 2.4 Sample size

Data was collected from eight Pac-12 athletic departments with an estimated NCAA DI student-athlete population *n* = 5,410 or about 69% of the estimated total Pac-12 population (N ~ 7,800). For the present study, based on N ~ 7,800, we used a confidence level of 95% with a margin of error of 5% and estimated that at least 50% of athletes use a third-party testing system while purchasing nutritional supplements, which resulted in a minimum number of 367 participants needed. An oversampling procedure was used to reduce self-recruitment bias and increase the generalization of findings while targeting an equal sex distribution. Therefore, this calculation was inflated by 1.5, targeting an equal number of men and women, resulting in a total of at least *n* = 560 athletes to recruit. To be able to recruit this number of student-athletes, an average participation of slightly over 10% for each athletic department was anticipated and maximal recruitment was capped by the total number of incentives at 13% (*n* = 725).

### 2.5 Statistical analysis

The demographics and descriptive data for relevant questionnaire sections are reported as percentages (%) and frequencies (n). When examining nutritional supplement use frequencies, products classified as “sport foods” (e.g., milk/flavored milk) were removed. To confirm the initial predictive model as reported previously (15) a cross-validation was performed to test the previously created algorithm on a new dataset. The purpose of this approach was to evaluate the generalizability of the initial algorithm and obtain new model fit indices that are less biased from overfitting that frequently occurs when assessing a model on the same dataset from which it was created. Based on the outputs from applying the initial algorithm to the new data, calculated predictions of the likelihood of TPT product use categorized in risk behavior score quadrants (i.e., 0–19%, 20–39%, 40–59%, 60–79%, 80–100%) that were crossed with consistent vs. inconsistent TPT supplement use to assess potential cut-off values in determining low vs. high risk. These tables were used to determine the best fit for the model, and Chi-Square analysis was used to determine statistical significance of the association between likelihood of TPT product use and observed TPT product use. Finally, the fit of the model was assessed by calculating the area under the curve (AUC), as well as true positive (TP), false positive (FP), false negative (FN), true negative (TN) scores. These values were used to calculate sensitivity (TP/TP + FN) and specificity (TN/TN + FP), as well as positive predictive value (PPV = TP/TP + FP) and negative predictive value (NPV = TN/TN + FN). Significance was set for *p* < 0.05 if not described differently.

Logistic regression analysis was used for explorative modeling to identify new variables associated with TPT supplement use that were not present in the initial predictive model created on the CPSDA dataset. The combined variables were then used to predict TPT supplement use to create and assess a new model based on the current Pac-12 dataset.

## 3 Results

### 3.1 Demographics and response

The total sample included 725 NCAA DI student-athletes, of which 50% were female (*n* = 362), and with an age of 20 ± 1.5 years, representing 24 sports (Table 1). Nearly all student-athletes (91%, *n* = 662) reported the use of at least one, but often more, nutritional supplements, not including sport foods, such as sports drinks and protein shakes and other products. A total of 56% (*n* = 405) of the athletes reported using one or more of their supplements TPT, but only 27% (*n* = 194) reported TPT supplement use (for all supplements, excluding sports foods, as listed in the Materials and methods section) consistently. Approximately 77% (*n* = 508) of the total sample reported recognizing at least one TPT organization logo.

### 3.2 Validation dataset

**Figure 1:**
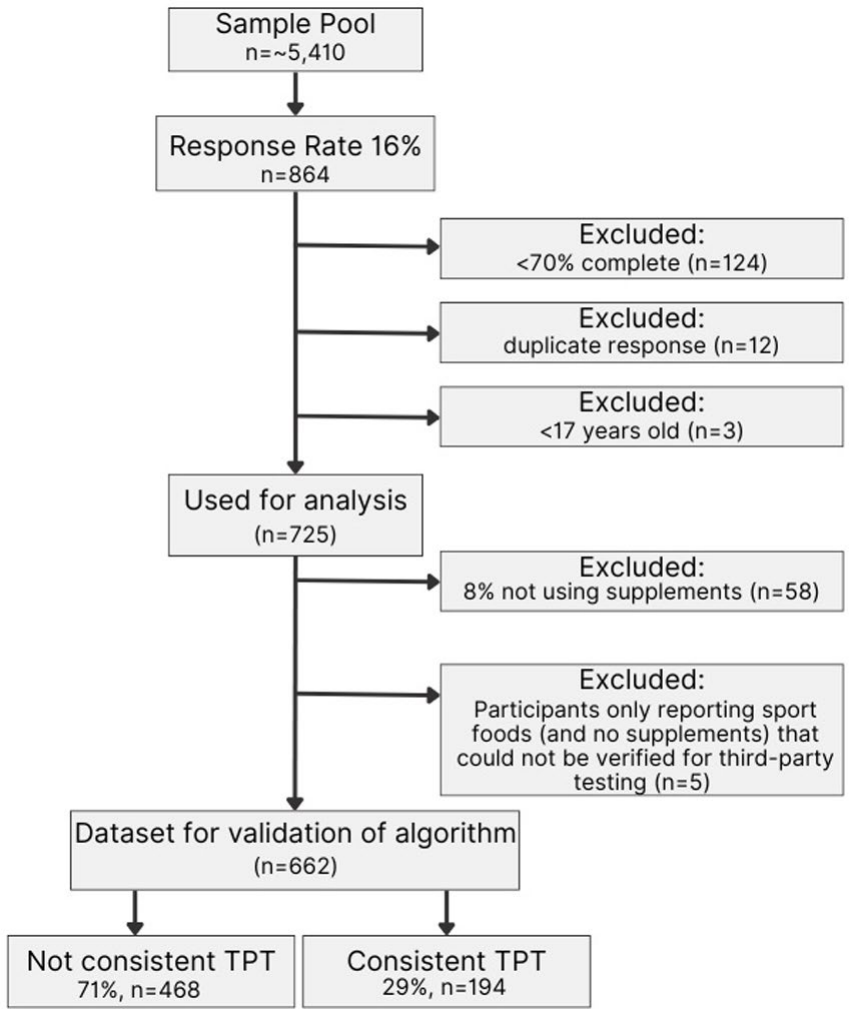
Consort diagram. The figure shows the response rate of NCAA DI student-athletes, the reported use of nutritional supplements, and the number of responses that were excluded before performing the cross-validation. TPT: Third-party-tested.

After removing nutritional sport foods (including sports drinks, sports bars, chocolate [flavored] milk, recovery drinks, energy drinks, and energy gels or chews) an additional 5 athletes were categorized as “non-users,” producing a total of 662 athletes which were used in the validation of the algorithm. Out of *n* = 662, a total of 29% (*n* = 194) consistently used TPT vs. 71% (*n* = 468) inconsistently used TPT (Figure 1).

### 3.3 Cross-validation

A total of *n* = 662 participants (all supplement users) were included to assess the fit of the previously obtained S3 algorithm, developed based on the initial responses on *n* = 320 athletes in a previous study (15), to classify a high vs. low risk of not using TPT supplements. Area under the curve (AUC) was calculated to determine the accuracy of the model on the new validation dataset.

#### 3.3.1 Algorithm variables using cross-validation data

As shown in Table 2, out of the 662 athletes, more than half (54%, *n* = 358) reported being familiar with the substances that may occur in nutritional supplements and that are found on the WADA banned substances list. Nearly the same number of athletes reported knowing where to find and order third-party tested supplements (53%, *n* = 353). Nearly 22% (*n* = 144) of these athletes do not search for information regarding nutritional supplements and sports foods and the majority of the athletes (62%, *n* = 413) reported that they discuss all supplement choices with their athletic departmental sports registered dietitian. Less than half of the surveyed athletes (44%, *n* = 289) reported that their decision to purchase a nutritional supplement was made with the advice of a friend, family, or a teammate, and 45% (*n* = 296) reported purchasing supplements outside of what is provided by their athletic department. Approximately half the athletes reported the use of multivitamin and mineral supplements (51%, *n* = 339). Very few athletes reported the use of a weight gainer (5%, *n* = 35), whereas 54% (*n* = 355) use caffeine. Finally, roughly one third of the athletes reported the use of creatine (30%, *n* = 200).

#### 3.3.2 Confirming a 60% cut-off for low vs. high risk of not using TPT using cross-validation data

Risk behavior score quadrants were plotted against consistent vs. inconsistent TPT use to evaluate the best-fit cut-off value to determine low vs. high risk, as shown in Table 3. This table shows that the original discriminatory cut-off was the best fit to classify low vs. high risk, resulting in a cut-off between quadrants three and four at the ≥60% mark.

**Table 3:** Algorithm-based risk behavior score for using vs. not using TPT quadrant against consistent vs. inconsistent TPT-use analysis for *n* = 662 NCAA Division I collegiate athletes.

	Risk behavior score quadrants: 1–5 (with % indicating algorithm outcome)					
TPT-use	1 (0–19%)	2 (20–39%)	3 (40–59%)	4 (60–79%)	5 (80–100%)	Total
Consistent (%, n)	0%	4%	7%	12%	6%	29%
	3	28	46	80	37	194
Inconsistent (%, n)	0%	1%	9%	30%	30%	71%
	1	7	62	201	197	468
Total* (%, n)	<1%	5%	16%	42%	35%	100%
	4	35	108	281	234	662

Chi-square analysis for risk quadrant analysis for consistent vs. inconsistent TPT-use was significantly different (χ^2^ (4) = 61.26, *p* < 0.001) suggesting a good discriminatory ability for the risk behavior score. *Total does not add up to 100% as all the percentages in the quadrants round down resulting in losing a full percent.

#### 3.3.3 Categorized results low vs. high risk of not using TPT supplements using cross-validation data

As shown in Table 4, the original S3 algorithm was able to identify significantly different responses (χ^2^ (1) = 15.95, *p* < 0.001), indicating a strong relationship between the risk groupings and product use outcomes. The screener had a fair sensitivity: the test’s ability to designate an individual with a negative outcome, as it classified 68% of the student-athletes not consistently using TPT accurately in the ≥60% high-risk behavior score group. Despite the test’s high sensitivity, the test had a low specificity (51%), as less than half of the consistent TPT users were correctly classified in the <60% low-risk behavior score group.

**Table 4:** Absolute and relative categorized screener-based answers for consistent/inconsistent TPT use vs. algorithm-based low/high-risk behavior toward TPT supplement use in *n* = 662 NCAA DI collegiate athletes.

	<60% algorithm score “Low risk”	≥60% algorithm score “High risk”	Total
Consistent TPT-use, % (n)	14% (95)	15% (99)	29% (194)
Inconsistent TPT-use, % (n)	23% (152)	48% (316)	71% (468)
(sub)Total, % (n)	37% (247)	63% (415)	100% (662)
Consistent TPT-use (Positive, n)	TN	FP	Specificity
	95	99	51%
Inconsistent TPT-use (Negative, n)	FN	TP	Sensitivity
	152	316	68%

Chi-square analysis for consistent/inconsistent TPT-use vs. algorithm-based low/high-risk behavior toward TPT supplement use was significantly different (χ^2^ (1) = 15.95, *p* < 0.001) suggesting a good discriminatory ability for the selected ≥ 60% risk behavior score cut-off. FP, False Positive; TN, True Negative; FN, False Negative; TP, True Positive.

#### 3.3.4 Area under the curve (AUC) using the validation data

**Figure 2:**
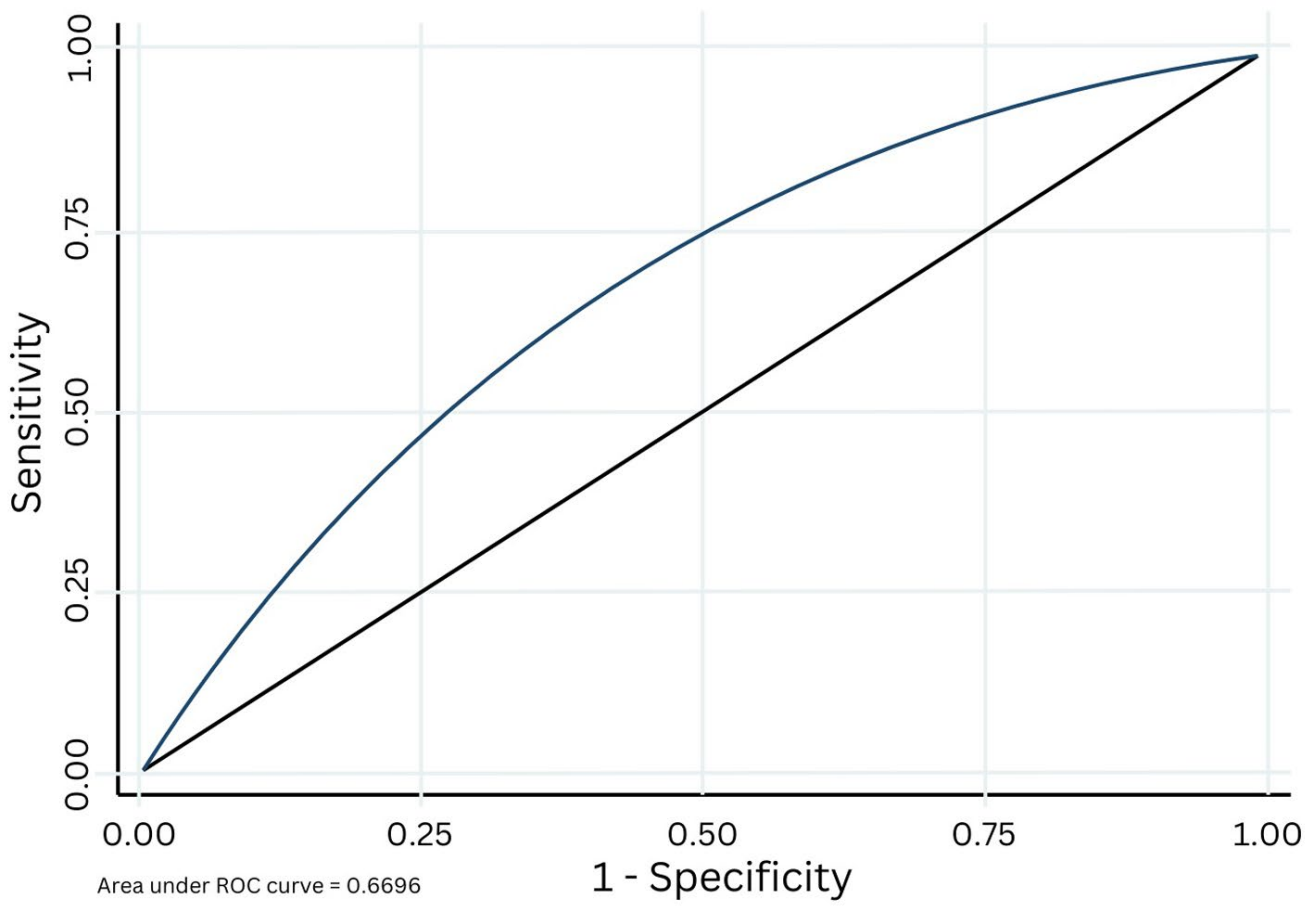
Area under the receiver operating characteristic (ROC) curve based on the cross validation of the original S3 algorithm applied to the current datase.

The AUC for the original S3 algorithm predicting inconsistent TPT product use on the new, Pac-12 data was 0.67 (Figure 2) indicating a moderate fit, with a positive predictive value (PVV) of 0.77, and negative predictive value (NPV) of 0.52.

### 3.4 Exploratory modeling for additional predictors

A stepwise logistic regression was run to create a predictive model based on the current dataset to see if additional predictor variables outside of those identified in the S3 algorithm would be related to use of TPT products. The *p* value threshold for variable entry was set at 0.25 and for variable retention in the final, restricted model was set at 0.10. These thresholds were selected to reduce the likelihood of a Type II error in an exploratory analysis with a small to moderate sample size. Input variables were the questions on the screener, and the outcome variable was a binary response of whether or not the respondent uses TPT supplements consistently. The predictive quality of the model was assessed by the Wald chi-square statistic for each question and the AUC for the full model. The LOGISTIC procedure in SAS 9.4 was used for model development. The final predictive model with significant variables is presented in Table 5.

**Table 5:** Outcome of exploratory analysis based on the current dataset to identify new potential predictors of third-party-tested (TPT) supplement use, including the prevalence per outcome for *n* = 662 NCAA Division I collegiate athletes.

Variable	Prevalence (% yes)	DF	Estimate	Standard error	Wald Chi-Square	Pr > Chi-Square
Intercept	–	1	0.54	0.41	1.71	0.19
**WADA Familiarity**: Are you familiar with banned substances that may occur in nutritional supplements listed on the WADA (world anti-doping agency) list? (Select only one – yes/no)	54%	1	0.37	0.14	6.41	**0.01**
**Recognizing at least one TPT organization logo:** I check all boxes of third-party testing systems icons that you recognize from products that you have used during the last 12 months. (Check all that apply)	77%	1	−0.68	0.29	5.57	**0.02**
**Having at least one name, image, likeness (NIL) deal**: Check all that apply concerning your athlete status. (Check all that apply)	15%	1	−0.60	0.27	4.94	**0.03**
**Discussing supplement choices with RD**: I discuss all my supplement choices with the Athletic Departmental Sports RD. (Only select one – yes/no)	62%	1	−0.47	0.19	6.16	**0.01**
**Advice from others**: I’ve decided to purchase one or more supplements as a result of the advice of family, friends, or teammates. (Only select one – yes/no)	44%	1	0.41	0.16	6.25	**0.01**
Please check all of the following nutritional supplements you have used during the last 12 months. (Check all that apply)						
**Multivitamin**: (Multivitamin and mineral supplement checked)	51%	1	0.77	0.22	12.35	**0.0004**
**Caffeine**: (Caffeine checked)	54%	1	0.91	0.22	16.83	**<0.0001**
**Creatine**: (Creatine checked)	30%	1	−0.78	0.24	10.79	**0.001**

These predictors were based on new predictive modeling based on the current dataset. DF = Degrees of freedom; Estimate = the weight and direction of the variable within the model; Wald Chi-Square = Test to determine if independent variables are significant for the model; Pr > Chi-Square = *p* – value that preferable was below or close to 0.10 as determined before the start of the modeling process.

The risk categories created from the algorithm constructed from the current dataset (Table 6) showed a statistically significant relationship with TPT product use (χ^2^ (1) = 48.57, *p* < 0.0001). Further, the test’s ability to designate an individual with a negative outcome (sensitivity) was high as it classified 85% of the student-athletes not consistently using TPT accurately in the ≥60% high-risk behavior score group. The test had a low specificity (40%), as half of the consistent TPT users were correctly classified in the <60% low-risk behavior score group. Finally, the area under the curve (AUC) for the cut-off value ≥60% suggesting a high risk for inconsistent TPT was 0.73, with a positive predictive value (PVV) of 0.77 and negative predictive value (NPV) of 0.50.

**Table 6:** Absolute and relative categorized screener-based answers for consistent/inconsistent third-party-tested supplement (TPT) use vs. algorithm-based low/high-risk behavior toward TPT supplement use in *n* = 662 NCAA Division I collegiate athletes.

	<60% algorithm score “Low risk”	≥60% algorithm score “High risk”	Total
Consistent TPT-use, % (n)	12% (77)	18% (117)	29% (194)
Inconsistent TPT-use, % (n)	10% (70)	60% (398)	71% (468)
(sub)Total, % (n)	22% (147)	78% (515)	100% (662)
Consistent TPT-use (Positive, n)	TN	FP	Specificity
	77	117	40%
Inconsistent TPT-use (Negative, n)	FN	TP	Sensitivity
	70	398	85%

Chi-square analysis for consistent/inconsistent TPT-use vs. algorithm-based low/high-risk behavior toward TPT supplement use was significantly different (χ^2^ (1) = 48.57, *p* < 0.001) suggesting a good discriminatory ability for the selected ≥ 60% risk behavior score cut-off. FP, False Positive; TN, True Negative; FN, False Negative; TP, True Positive.

## 4 Discussion

### 4.1 Recap of the study outcomes

This study confirms previous findings that confirmatory checks of self-reported third-party-tested (TPT) supplement use can help to accurately identify inconsistent TPT users. When testing the validity of the original algorithm on the current dataset, overall, the algorithm was confirmed to be able to identify different responses with high sensitivity but low specificity. When exploring additional predictors indicating TPT use using the current dataset with some new questions, the new algorithm showed a similar but lower sensitivity and higher specificity and higher area under the curve (AUC).

### 4.2 Cross-validation

When developing the original S3 algorithm, the initial training-data produced a reasonable AUC (0.78) with a high sensitivity (89%) and a low specificity (49%) (15). During that study, a preliminary cross-validation was also performed on a small subsample of the dataset (*n* = 34), revealing a lower AUC (0.61), sensitivity (83%), and specificity (40%) (15). The current study allowed for cross-validation of the algorithm with a much larger dataset (*n* = 662), revealing a moderate AUC (0.67), with a sensitivity of 68%, and a specificity of 51%. The current study therefore reported a higher AUC than the cross-validation on the small subsample of the data that were used to develop the S3 algorithm. The sensitivity of 68% indicated that the algorithm specifically classifies the inconsistent TPT supplement users correctly. It is important to note, when the algorithm was initially applied on its own data, which also were used for its development, this likely inflated the accuracy of the algorithm, warranting the current cross-validation using an independent dataset (20).

### 4.3 Predictors of TPT use and further explorative modeling

Of the ten predictive variables that originally constructed the S3 algorithm ([1] WADA familiarity; [2] knowing where to find and order TPT supplements; [3] discussing supplement choices with a sports RD; [4] not searching for information; [5] purchasing products outside of the supplements provided by the athletic department; [6] deciding to purchase supplements based on the advice of others, such as family, friends or teammates; and using [7] multivitamins, [8] weight gainer, [9] creatine, and [10] caffeine) six were found to be significantly related to this current dataset. Additionally, the exploratory model identified two new variables as significantly related to the prediction of consistent vs. inconsistent use of TPT supplements while also reporting six of the previously identified variables. These new variables include (1) recognizing at least one TPT organization logo and (2) having at least one name, image, likeness (NIL) deal, and were added to the most recent questionnaire. These two new variables may provide further insight in the future into what may be driving athlete choices.

Third-party testing organizations are a vital aspect of reducing supplement contamination risk as these organizations provide quality assurance for athletes who use supplements. The safety of supplements is never fully assured; however, the use of a TPT product, specifically those tested for banned substances in sport, can help reduce the risk of inadvertent doping (21). The majority of athletes believe that contaminated supplements can lead to positive doping tests (22), however, consistency is low with only about 38–57% reporting every supplement they used as being tested (2, 14). Approximately 66% of athletes report recognizing at least one logo that references TPT organizations and 90% believe that it is essential to know if a supplement has been tested, but the concern still lies in the prevalent non-compliance (2, 14). A reason for athletes not consistently reporting the use of third-party tested supplements could be that they feel that the type and/or quality of product they are looking for is not available, at the same time this is something that normally would be covered by having targeted conversations about the athletes’ nutritional supplement selection with the designated sports RD. It has been seen in high school athletes that education, which includes the safety and use of TPT products, may help to influence athlete choices and encourage the use of safer supplementation (i.e., TPT products) (18). While educational programs may help to influence athlete decisions and encourage safer supplement use in younger athletes, no clear relationship has previously been reported between TPT logo recognition and the consistent use of TPT products at the collegiate level (14). Although simplified stratifications, including TPT logo recognition, have not previously identified a relationship with TPT use, the stepwise comparison performed as an exploratory analysis in the present study identified that in conjunction with other predictive variables, a relationship between the ability of an athlete to recognize TPT organization logos and the consistent use of TPT products may exist and may require further examination in this population.

Prior to 2021, collegiate athletes were barred from obtaining benefits, monetary or otherwise for the use of their name, image and likeness (23). There are many ways athletes may have been affected in recent years since the rule change, including pressure to use supplements to increase performance or recovery and possibly pressure to purchase or use specific supplements not provided by the department per their deals. Since this rule changed, many student athletes have taken advantage of these opportunities. Nearly 17% of DI student athletes are reported to have had NIL deals in 2022 (24). It has been reported that one of the key dimensions of an NIL deal is the athlete’s athletic performance (25). The added pressure of these deals may increase pressure on athletes to find ways to increase or maintain their athletic performance (26). It has been reported that most athletes (58–64%) report performance enhancement as the driving reason for supplement use (2, 27).

Ergogenic aids are produced and marketed as a performance enhancement or an athlete’s recovery after training or competition (28). Whether these products truly enhance performance or speed recovery is mostly unknown; however, there are some products, such as caffeine, creatine, dietary nitrate, beta-alanine, and sodium bicarbonate, which the literature strongly supports (2). The concern behind the use of ergogenic aids is the increased risk of inadvertent doping. Ergogenic aids are commonly reported to be laced with contaminants including various anabolic androgenic steroids, ephedrine, methamphetamine analogs and extreme levels of caffeine (29–31). Athletes who choose to use ergogenic aids should always be utilizing products which have been tested by a TPT organization that tests for banned substances and label accuracy. Per the NCAA bylaws, athletic departments are allowed to provide specific nutritional supplements; however, there are restrictions to the extent and types of products departments are allowed to provide (32). Many athletes may purchase outside of what is provided by the department (14) and some may be provided with supplements from sponsors (33, 34). As such, it is important that athletes be taught the importance of using tested products and the risks of using products that have not been tested for banned substances. As the popularity of NIL grows, it is likely that sponsorships from supplement companies may grow as well; however, there is no reported connection currently between NIL deals and supplement use (35). Future studies looking into the decision-making processes occurring how and why athletes choose specific nutritional supplements are vital to better understand how practitioners can increase athlete safety. In addition, research is needed to better understand how certain variables like NIL, product type, −quality, and -efficacy, as well as third-party testing availability may affect an athlete’s decision to use or not use specific nutritional supplements.

Although these new variables (i.e., TPT logo recognition and NIL) provide an interesting look into the habits and characteristics of college athletes who use supplements, the importance of the variables used in the S3 screener are invaluable. The variables WADA familiarity; knowing where to find and order TPT supplements; discussing supplement choices with a sports RD; and using multivitamins, weight gainer, and creatine are associated with consistent use of TPT supplements, while not searching for information; purchasing products outside of the supplements provided by the athletic department; deciding to purchase supplements based on the advice of others, such as family, friends or team mates; and using caffeine are related to inconsistent TPT supplement use. Because of the prevalent risk of contaminated substances, it is ever important to minimize the risky behaviors of athletes. One method to do so is to survey these athletes, using tools like the S3 screener. The addition of these newly identified variables to the screener may add another layer for sport professionals to continue to screen their athletes. Further, this will allow for additional future cross-validation to determine if these variables also should be included in the future S3 algorithm. In the meantime, these variables are available as part of the screener for practitioners to be used (Supplementary File 3). Practically, this screener can be useful for health professionals who work with athletes, such as sports RDs and athletic trainers to better understand nutritional supplement use and behavior of their athletes. The screener can help to categorize athletes in certain groups, such as consistent TPT users, inconsistent TPT users, and non-users of supplements. In addition, the S3 tool, will help to gain understanding of some of the behaviors, such as an athlete’s familiarity with the list of banned substances from WADA, their knowledge of where to find and order TPT supplements and their open discussion with an RD regarding their supplement use which are positively associated with using TPT nutritional supplements, vs. an athlete not searching for information regarding supplements and sports foods, taking supplement advice from resources such as friends, family or teammates and purchasing supplements outside of those which are supplied by their respective athletic department that are negatively associated with the use of TPT nutritional supplements. This allows for targeted education, focusing on improving TPT nutritional supplement use compliance, and reducing high risk behavior. Finally, when health professionals include the screener in their dietary monitoring on an annual basis nutritional supplement use and behavior can be followed over time, which may help to show the impact of education efforts, and it can help to justify budget decision focusing on athlete safety within the organization.

### 4.4 Strengths and limitations

The strength of this study is led by the size of the data collected. In total, 864 responses were collected and after data cleaning and removal for completion, a 12% response rate was obtained. Another strength of the study was anonymity. All responses collected were anonymous, which theoretically should have increased honesty of responses and reduced fear of consequences when reporting behavior that is not in line with athletic department policy (36). In addition to these strengths, the generalizability to NCAA DI athletes should be fairly reasonable, as responses collected were nearly split perfectly between male and female responses and included a large variety of NCAA DI sports.

The study was not without its limitations. Although athlete responses were not connected to their personal identifiable information, it is known that self-reported data can contain errors, such as over or under estimation (37). As such, the results may not be as accurate. At the same time, the (type of) questions asked, have been repeatedly used in other investigations (14, 15) and therefore comparison with other resources should allow to identify important differences and shifts with other investigations. Additionally, the sample was collected from one single NCAA conference, the Pac-12, covering mainly the western United States, potentially limiting generalization on a national level. While the sample pool included three schools, which were also included in the dataset that created the original algorithm, the possibility of cross-over exists; however, after examining responses, it is estimated that only one duplicate response was recorded (0%). Finally, similar to the original S3 algorithm, data are reported from DI athletes, and it has been reported that NIL deals are significantly more prominent in DI athletics than in DIII (35) so it may be difficult to generalize these data to other levels. As the impact of NIL is still increasing, the relation between NIL and TPT supplement use needs to be further investigated.

## 5 Conclusion

The current study shows that cross-validation of the S3 algorithm to predict TPT supplement use by collegiate athletes reports a lower accuracy than previously reported. At the same time the 60% cut-off to predict consistent vs. inconsistent TPT use has been confirmed. Despite the lower accuracy, the S3 algorithm still classifies the majority of inconsistent TPT supplement users in the high-risk group. Finally, exploratory analysis revealed that TPT logo recognition and NIL were related to consistent TPT use, warranting including the variables (and their associated questions) in the S3 screener without directly contributing to the algorithm. This information on logo recognition and NIL may be beneficial for health professionals who work with athletes, such as sports RDs and athletic trainers, in their efforts to minimize unsafe supplement use in their athletes.

## Data Availability

The raw data supporting the conclusions of this article will be made available by the authors, without undue reservation.
