# Supplementary material for: Cross-validation of the safe supplement screener (S3) predicting consistent third-party-tested nutritional supplement use in NCAA Division I athletes
Source: Front Nutr. 2025 Jan 15;11:1519544. doi: 10.3389/fnut.2024.1519544 (PMC11792857; doi:10.3389/fnut.2024.1519544)
Supplement: Supplementary file 1 [file Data_Sheet_1.zip › Supplementary Material File 2.DOCX]

Safe Supplement Screener (S3)

**Introduction**

This is a short screening questionnaire about your nutritional supplement use.
It will take 2-3 minutes to fill out this questionnaire.

Please read this text below to make sure you understand what we will be questioning.

The following definitions are used for supplements:

 **Nutritional supplements:** The combination of all dietary supplements, sports foods, and

ergogenic supplements.

 **Dietary supplements:** Are considered vitamins, minerals, and essential fatty acids.

 **Sports foods:** Are considered sports drinks, protein shakes, and sports bars.

 **Performance-enhancing (ergogenic) supplements**: Are considered supplements that most

of the time are not provided by your Athletic Department or Sport Organization (such as

creatine) that go with a performance-enhancing claim. Keep in mind that an ergogenic

supplement's definition is based on its claim and not necessarily on the evidence of its efficacy.

**General questions**

Q1 Please write your first and last name.

________________________________________________________________

Q2 What is your primary sport? (Define sport and if this is a Men [M] or Women [W] roster)

________________________________________________________________

**Questions about your supplement behavior**

Q3 Are you familiar with banned substances that may occur in nutritional supplements listed on the WADA (world anti-doping agency) or your own sport specific association? (Select only one)

- Yes
- No
- Not Sure

Q4 I know where to find and order third-party tested supplements. (Only select one)

- Agree
- Disagree
- Not Sure

Q5 I discuss all my supplement choices with the Athletic Departmental Sports RD. (Only select one)

- Agree
- Disagree
- Not Sure

Q6 If a teammate uses a supplement, I am more likely to try it as well. (Only select one)

- Agree
- Disagree
- Not Sure

Q7 I've decided to purchase one or more supplements as a result of the advice of family, friends, or teammates. (Only select one)

- Agree
- Disagree
- Not Sure

Q8 Do you purchase or use nutritional supplements outside what is provided by your Athletic Department? (Only select one)

- Yes
- No

Q9 Where do you go to look for information on nutritional supplements and sports foods? (Check all that apply)

- Scientific search engines (i.e., PubMed, Google Scholar)
- Regular internet search engines (i.e., Google, Amazon)
- Podcasts, videos or blogs
- I do not search for information on my own

Q10 Where do you receive your nutritional supplements from? (Check all that apply)

- Brick and mortar store, go to Q10.1
- Online/ecommerce, go to Q10.1
- Directly from brand, go to Q10.1
- Athletic Department, go to Q10.1
- I do not currently use nutritional supplements, go to Q11

Q10.1 If you use supplements or sports foods that your Athletic Department is **not providing**, who is purchasing them? (Check all that apply)

- I am
- Parents
- Other

**Nutritional Supplement Use**

Q11 Please check all of the nutritional supplements you have used during the last 12 months and identify if they were third-party tested (Q12).
(Check all that apply, supplements are listed in alphabetical order)

Before you fill out the right side of the table please read the information below:
**"Third-party testing is when an organization that is not the supplement company itself (hence, the third-party) evaluates a dietary supplement and vouches for its quality,” (United States Anti-Doping Agency, 2022). There are multiple third-party testing organizations that work to certify supplements. Athletes should preferably select these third-party tested products from organizations that test supplements for banned substances."**

| Check all supplements that you used during the last 12 months | | Identify for each supplement you checked on the left if it was third-party tested during the last 12 months. | | |
| --- | --- | --- | --- | --- |
|  |  | Yes | No | Not Sure |
| O | Aswagandha (or also known as: Indian ginseng, poison gooseberry, winter cherry) (1) | O | O | O |
| O | BCAA (2) | O | O | O |
| O | Beta-Alanine (3) | O | O | O |
| O | Caffeine (4) | O | O | O |
| O | CBD (Cannabidiol) (5) | O | O | O |
| O | Combination of vitamins (6) | O | O | O |
| O | Combination of minerals (7) | O | O | O |
| O | Chocolate milk (or other flavored milk option with sugar added like strawberry milk) (8) | O | O | O |
| O | CLA (9) | O | O | O |
| O | Co-enzyme Q10 (also known as: CoQ10) (10) | O | O | O |
| O | Collagen (11) | O | O | O |
| O | Colostrum (12) | O | O | O |
| O | Creatine (13) | O | O | O |
| O | Deer Antler Velvet extract (14) | O | O | O |
| O | Dietary nitrate (for example but not limited to beetroot juice) (15) | O | O | O |
| O | Dendrobium (16) | O | O | O |
| O | Energy drink (for example but not limited to Red Bull, Monster and other energy drinks) (17) | O | O | O |
| O | Energy gel or chewies (for example but not limited to Gu, Gatorade, and Powerbar) (18) | O | O | O |
| O | Ephedra (19) | O | O | O |
| O | Exotic berries (for example but not limited to acai, and goji) (20) | O | O | O |
| O | Fish oil/ essential fatty acids (21) | O | O | O |
| O | Fenugreek (or also known as: Methi) (22) | O | O | O |
| O | Glucosamine (23) | O | O | O |
| O | Glutamine (24) | O | O | O |
| O | Glycerol (25) | O | O | O |
| O | Herbs (such as: echinacea, ginseng, and ginkgo biloba or others herbs) (26) | O | O | O |

| Check all supplements that you used during the last 12 months | | Identify for each supplement you checked on the left if it was third-party tested during the last 12 months. | | |
| --- | --- | --- | --- | --- |
|  |  | Yes | No | Not Sure |
| O | HMB (27) | O | O | O |
| O | Kava (or also known as: kava kava, awa, ava, yaqona, yagona, seka, malok or malogu) (28) | O | O | O |
| O | L-carnitine (29) | O | O | O |
| O | Leucine (30) | O | O | O |
| O | Longjack (31) | O | O | O |
| O | Maca root powder (32) | O | O | O |
| O | Medium-chain triglycerides (MCT) (33) | O | O | O |
| O | Methylliberine (or also known as; dynamine, tetramethylurate or tetramethyluric acid) (34) | O | O | O |
| O | Multivitamin and mineral supplement (35) | O | O | O |
| O | Muscle cramp relievers (such as supplements based on: pickle juice, menthol, and sour, bitter or spicy substances) (36) | O | O | O |
| O | Pre-workout supplement (37) | O | O | O |
| O | Probiotics (38) | O | O | O |
| O | Protein shake (39) | O | O | O |
| O | Phyllanthus (or leafflower) (40) | O | O | O |
| O | Quercitine (41) | O | O | O |
| O | Recovery drink (for example but not limited to Muscle Milk, and Rocking Refuel) (42) | O | O | O |
| O | Ribose (43) | O | O | O |
| O | SARMs (such as: Ostarine, Andarine, Ligandrol (LGD-4033), and RAD140) (44) | O | O | O |
| O | Single mineral (for example but not limited to calcium, iron, magnesium, and zinc) (45) | O | O | O |
| O | Single vitamin (for example but not limited to vitamin C, vitamin D, and vitamin E) (46) | O | O | O |
| O | Sodium bicarbonate (47) | O | O | O |
| O | Sports bar (for example but not limited to an energy bar, and protein bar) (48) | O | O | O |
| O | Sports drink (for example but not limited to Powerade, and Gatorade) (49) | O | O | O |
| O | Tart Cherry (or other cherry varieties) (50) | O | O | O |
| O | Tribulus terrestris (51) | O | O | O |
| O | Weight gainer (52) | O | O | O |
| O | Other, go to Q11.1 (53) | O | O | O |
| O | None of the above (54) | O | O | O |

Q11.1 Please write down any other supplements that you have used in the last 12 months that were not listed just above.

________________________________________________________________

________________________________________________________________

**Potentially relevant questions to be added to the screener**

Q13

Check all boxes of third-party testing systems icons that you recognize from products that you have used during the last 12 months. (Check all that apply)


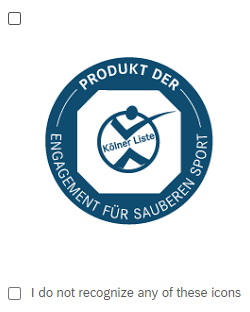

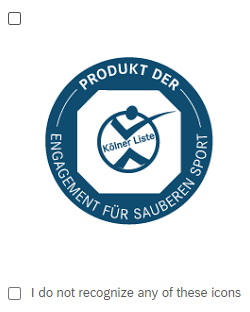

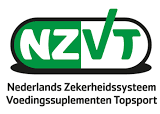

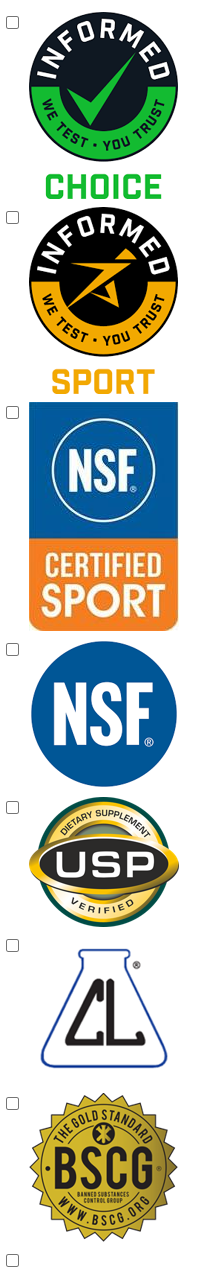

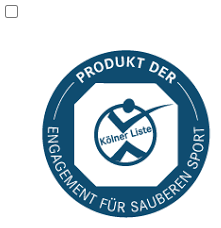

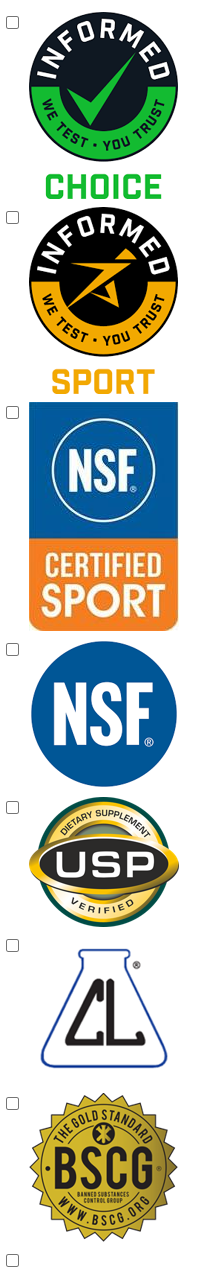


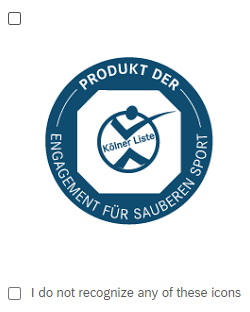


Q14
Check all that apply concerning your athlete status. (Check all that apply)

- Carded athlete
- Part of a national doping pool
- Member of a national team or selection
- Student-athlete at a US collegiate athletic department
- Student-athlete not at a US collegiate athletic department
- Professional athlete
- I have one ore more Name Image and Likeness (NIL) deals

We thank you for your time spent taking this survey. Please hand over the questionnaire to your point of contact!

| **More information about the development of this screener and its additional materials can be found in:**  Wardenaar FC, Schott KD, Seltzer RGN and Gardner CD (2024) Development of a  screener to assess athlete risk behavior of not using third-party tested nutritional supplements. Front. Nutr. 11:1381731. doi: 10.3389/fnut.2024.1381731  Supplementary materials (i.e. Qualtrics file, and excel file with stepwise explanation how to calculate the risk behavior score) can be downloaded at:  <https://www.frontiersin.org/journals/nutrition/articles/10.3389/fnut.2024.1381731/full#supplementary-material> |
| --- |
