# Supplementary material for: Cross-validation of the safe supplement screener (S3) predicting consistent third-party-tested nutritional supplement use in NCAA Division I athletes
Source: Front Nutr. 2025 Jan 15;11:1519544. doi: 10.3389/fnut.2024.1519544 (PMC11792857; doi:10.3389/fnut.2024.1519544)
Supplement: Supplementary file 1 [file Data_Sheet_1.zip › Supplementary Material File 1.DOCX]

Nutritional Supplement Questionnaire

Start of Block: Introduction

Q1 **Introduction**

Q2 Dear Athlete,

 Thank you for being part of this study in which we investigate the knowledge, motivations and attitudes, and barriers associated with safe nutritional supplement use in a collegiate athletic population. It will take 15-20 minutes of your time, depending on the answers that you provide.

 The questionnaire covers multiple topics like: general questions, nutritional supplement use, knowledge about supplements, attitudes and barriers towards nutritional supplement use.

 The following definitions are used for supplements in this questionnaire:

 **Nutritional supplements:** The combination of all dietary supplements, sports foods, and ergogenic supplements.

 **Dietary supplements:** Are considered vitamins, minerals, and essential fatty acids.

 **Sports foods:** Are considered sports drinks, protein shakes, and sports bars.

 **Performance-enhancing (ergogenic) supplements**: Are considered supplements that most of the time are not provided by your Athletic Department or Sport Organization (such as creatine) that go with a performance-enhancing claim.
 Keep in mind that an ergogenic supplement's definition is based on its claim and not necessarily on the evidence of its efficacy.

 We are aiming to recruit 10-12% of the athlete total from your organization, after this the questionnaire will be closed.
 To receive your incentive, you will need to provide a complete questionnaire, followed by your personal information (first and last name, and a valid university email address) that you can provide in a separate follow-up questionnaire. As soon as we receive your full response we will start processing the incentive. Normally, you will receive your incentive within 7 work days after filling out the questionnaire. Make sure to claim the gift card ($50.00) after you receive it.

 Thank you for your time.

| Page Break |  |
| --- | --- |

Q3 **Signing informed consent (study clarifaction removed)**

Q5 To ensure your personal information is collected separately from the answers you provide you will be directed to another questionnaire at the end of this survey allowing you to fill out your first and last name, and email address.

Identify below if you consent to participate in this study:

- I consent to participate (please forward to the next part of the survey) (1)

| Page Break |  |
| --- | --- |

Q6 The questionnaire is broken up into small sections to increase readability. Make sure you read each question carefully before you insert your answer. You will not be able to correct answers after you have pushed the "next" button

End of Block: Introduction

Start of Block: General questions

Q7 **General questions**

| 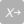 |
| --- |

Q8 What is your Athletic Department?

- Arizona State University (1)
- Stanford University (3)
- University of Utah (5)
- University of California Berkeley (7)
- University of Colorado Boulder (8)
- Washington State University (9)
- University of Washington (10)
- University of Arizona (11)

| Page Break |  |
| --- | --- |

Q110 Are you a freshman?

- Yes (1)
- No (7)

| Page Break |  |
| --- | --- |

Display This Question:

If Are you a freshman? = No

Q111 Did you transfer from another University?

- No (1)
- Yes, from another DI athletic program (4)
- Yes, from another athletic program other than DI (such as DII, DII, or JU-CO) (2)

| Page Break |  |
| --- | --- |

|  |
| --- |

Q9 What is your primary sport?

- Artistic Swimming (1)
- Baseball (2)
- Basketball (M) (3)
- Basketball (W) (4)
- Beach Volleyball (5)
- Cross Country (M) (6)
- Cross Country (W) (7)
- Fencing (M) (8)
- Fencing (W) (9)
- Field Hockey (M) (10)
- Field Hockey (W) (47)
- Football (11)
- Golf (M) (12)
- Golf (W) (13)
- Gymnastics (M) (14)
- Gymnastics (W) (15)
- Ice Hockey (16)
- Lacrosse (M) (17)
- Lacrosse (W) (18)
- Rifle (19)
- Rowing (M) (20)
- Rowing (W) (21)
- Rowing Lightweight (M) (48)
- Rowing Lightweight (W) (49)
- Rugby (M) (51)
- Rugby (W) (52)
- Sailing (M) (22)
- Sailing (W) (23)
- Skiing (M) (24)
- Skiing (W) (25)
- Soccer (M) (26)
- Soccer (W) (27)
- Softball (28)
- Swimming & Diving (M) (29)
- Swimming & Diving (W) (30)
- Squash (M) (31)
- Squash (W) (50)
- Tennis (M) (32)
- Tennis (W) (33)
- Track and Field (M) (34)
- Track and Field (W) (35)
- Triathlon (M) (45)
- Triathlon (W) (46)
- Volleyball (M) (36)
- Volleyball (W) (37)
- Water Polo (M) (38)
- Water Polo (W) (39)
- Wrestling (40)
- Cheerleading (M) (41)
- Cheerleading (W) (42)
- Dance (M) (43)
- Dance (W) (44)

| Page Break |  |
| --- | --- |

| 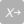 |
| --- |

Q10 What is your sex?

- Female (1)
- Male (2)
- Intersex (3)

| Page Break |  |
| --- | --- |

| 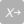 |
| --- |

Q101 For you only, are you Hispanic or Latino?

- Yes (1)
- No (0)

| Page Break |  |
| --- | --- |

Q102 Which of these categories best indicates your race? Answer for yourself.
(please select one or more)

- American Indian or Alaskan Native (1)
- Asian (2)
- Black or African American (3)
- Native Hawaiian or other Pacific Islander (4)
- White (5)

| Page Break |  |
| --- | --- |

|  |
| --- |

Q11 What is your current age? (years)

- 17 (17)
- 18 (18)
- 19 (19)
- 20 (20)
- 21 (21)
- 22 (22)
- 23 (23)
- 24 (24)
- 25 (25)
- 26 (26)
- 27 (27)
- 28 (28)
- 29 (29)
- 30 (30)
- 31 (31)
- 32 (32)
- 33 (33)
- 34 (34)
- 35 (35)
- 36 (36)
- 37 (37)
- 38 (38)
- 39 (39)
- 40 (40)
- 41 (41)
- 42 (42)
- 43 (43)
- 44 (44)
- 45 (45)

| Page Break |  |
| --- | --- |

| 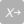 |
| --- |

Q12 Check all that apply concerning your athlete status.
(Check all that apply)

- Carded athlete (1)
- Part of a national doping testing pool (2)
- Member of a national team or selection (3)
- Student-athlete at a US collegiate athletic department (4)
- Student-athlete not at a US collegiate athletic department (5)
- Professional athlete (6)
- I have one or more Name Image and Likeness (NIL) deals (8)

End of Block: General questions

Start of Block: Information Sources

Q13 **Information sources**

| 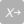 |
| --- |

Q14 Identify if you have received nutrition information, counselling or advice during the last 12 months from any of the people or professions below by tagging one or more options below. (Check all that apply)

- Sports Registered Dietitian/Nutritionist within the Athletic Department or Sport Organization (1)
- Sports Registered Dietitian/Nutritionist outside the Athletic Department or Sport Organization (2)
- I did not receive any nutrition information, counselling, or advice during the last 12 months (4)

| Page Break |  |
| --- | --- |

Display This Question:

If Identify if you have received nutrition information, counselling or advice during the last 12 mon... = Sports Registered Dietitian/Nutritionist within the Athletic Department or Sport Organization

Or Identify if you have received nutrition information, counselling or advice during the last 12 mon... = Sports Registered Dietitian/Nutritionist outside the Athletic Department or Sport Organization

Carry Forward Selected Choices from "Identify if you have received nutrition information, counselling or advice during the last 12 months from any of the people or professions below by tagging one or more options below. (Check all that apply)"

| 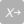 |
| --- |

Q15 Define the number of contact moments that you had with each professional during the last 12 months. (Select an answer for each horizontal row, select NA if non-applicable)

|  | 1-2 visits (1) | 3-6 visits (2) | 7-10 visits (3) | 11 or more visits (4) | NA (5) |
| --- | --- | --- | --- | --- | --- |
| Sports Registered Dietitian/Nutritionist within the Athletic Department or Sport Organization (x2) |  |  |  |  |  |
| Sports Registered Dietitian/Nutritionist outside the Athletic Department or Sport Organization (x3) |  |  |  |  |  |
| I did not receive any nutrition information, counselling, or advice during the last 12 months (x6) |  |  |  |  |  |

| Page Break |  |
| --- | --- |

Display This Question:

If Identify if you have received nutrition information, counselling or advice during the last 12 mon... = Sports Registered Dietitian/Nutritionist within the Athletic Department or Sport Organization

Or Identify if you have received nutrition information, counselling or advice during the last 12 mon... = Sports Registered Dietitian/Nutritionist outside the Athletic Department or Sport Organization

| 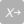 |
| --- |

Q16 Please check all types of information/topics that were addressed in these sessions/materials. (Check all that apply)

- Food first approach (1)
- Risk of nutritional supplement use, especially the ones not provided by the Athletic Department (2)
- Anti-doping education (3)
- Third-party testing (4)
- Performance-enhancing supplements (i.e. ergogenic aids) (5)

|  |
| --- |

- Sources that help to make informed decisions on the use of dietary supplements (6)
- Identification of doping related substances that may appear on a product label (7)

| Page Break |  |
| --- | --- |

Q17 Who is your preferred source within the Athletic Department to go to when you have questions about the use of nutritional supplements. (Select only one)

- Sports Dietitian (or sports RD) or dietitian (or RD) (1)
- Athletic Trainer (2)
- Strength & Conditioning Coach (3)
- Coach (4)
- Physician (5)
- Team manager (6)
- Team member (another athlete within your team or Athletic Department) (7)
- Athletic Director (8)

| Page Break |  |
| --- | --- |

| 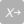 |
| --- |

Q18 Where do you go to look for information on nutritional supplements and sports foods?
(Check all that apply)

- Scientific search engines (i.e., PubMed, Google Scholar) (1)
- Regular internet search engines (i.e., Google, Amazon) (2)
- Podcasts, videos or blogs (3)
- I do not search for information on my own (4)

End of Block: Information Sources

Start of Block: Social Media

Q19 **Social Media**

| 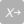 |
| --- |

20 Do you use the following types of social media?
(Select a score: No or Yes for all listed items)

|  | No (0) | Yes (1) |
| --- | --- | --- |
| Facebook (1) |  |  |
| Instagram / threads (4) |  |  |
| Snapchat (5) |  |  |
| YouTube (6) |  |  |
| X (FKA as Twitter) (7) |  |  |
| TikTok (8) |  |  |
| Pinterest (9) |  |  |

| Page Break |  |
| --- | --- |

Display This Question:

If Do you use the following types of social media? (Select a score: No or Yes for all listed items) [ Yes] (Count) >= 1

Carry Forward Selected Choices from "Do you use the following types of social media? (Select a score: No or Yes for all listed items)"

| 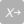 |
| --- |

Q21 How often do you check social media (even if you are logged in all day)?

|  | Not at all (1) | Every few days (2) | Once a day (3) | every few hours (4) | every hour (5) | every 30 minutes (6) | every 10 minutes (7) | every 5 minutes (8) |
| --- | --- | --- | --- | --- | --- | --- | --- | --- |
| Facebook (x1) |  |  |  |  |  |  |  |  |
| Instagram / threads (x4) |  |  |  |  |  |  |  |  |
| Snapchat (x5) |  |  |  |  |  |  |  |  |
| YouTube (x6) |  |  |  |  |  |  |  |  |
| X (FKA as Twitter) (x7) |  |  |  |  |  |  |  |  |
| TikTok (x8) |  |  |  |  |  |  |  |  |
| Pinterest (x9) |  |  |  |  |  |  |  |  |

| Page Break |  |
| --- | --- |

Display This Question:

If Do you use the following types of social media? (Select a score: No or Yes for all listed items) [ Yes] (Count) >= 1

| 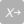 |
| --- |

|  |
| --- |

Q22 How much time do you spend using your combined social media on average per day?

- 5 minutes or less (1)
- 15 minutes (2)
- 30 minutes (3)
- 1 hour (4)
- 2 hours (5)
- 3 hours (6)
- 4 hours (7)
- 5 hours (8)
- 6 hours (9)
- 7 hours (10)
- 8 hours (11)
- 9 hours (12)
- 10 hours or more (13)

| Page Break |  |
| --- | --- |

Q24 What would be the best way for your athletic department to reach you with information related to banned substance education?
(Check all that apply)

- In person (including video call) (1)
- Per email (3)
- Per text (16)
- Via an app (14)
- Via social media (15)

End of Block: Social Media

Start of Block: Supplement Knowledge

Q25 **Nutritional Supplement Knowledge**

| 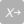 |
| --- |

26 The following are statements about athletes' needs for particular micronutrient supplements. (Please select Agree, Disagree, or Not Sure for each item)

|  | Agree (1) | Disagree (2) | Not Sure (3) |
| --- | --- | --- | --- |
| Vitamin C should be routinely supplemented by athletes. (1) |  |  |  |
| B vitamins should be taken when feeling low in energy during exercise. (2) |  |  |  |
| Salt tablets should be used by athletes that get a cramp. (3) |  |  |  |
| Iron tablets should be taken when a player feels extremely tired and is pale (4) |  |  |  |

| Page Break |  |
| --- | --- |

| 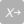 |
| --- |

Q27 The purity and safety of all supplements are tested before sale. (Select only one)

- Agree (1)
- Disagree (2)
- Not Sure (3)

| Page Break |  |
| --- | --- |

| 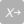 |
| --- |

Q28 Supplement labels may contain false or misleading information. (Select only one)

- Agree (1)
- Disagree (2)
- Not Sure (3)

| Page Break |  |
| --- | --- |

|  |
| --- |

Q29 The following statements are about the reported benefits of performance-enhancing supplements. (Please select Agree, Disagree, or Not Sure for each item)

|  | Agree (1) | Disagree (2) | Not Sure (3) |
| --- | --- | --- | --- |
| Creatine reduces the perceived effort of exercise by acting on the central nervous system. (1) |  |  |  |
| Caffeine improves the efficiency of muscles at a given rate of oxygen delivery. (2) |  |  |  |
| Beetroot Juice (nitrates) decrease muscle breakdown and reduce muscle soreness (3) |  |  |  |
| Beta-Alanine produces carnosine, a protein that can buffer (“soak up”) acid by-products produced during high intensity activity. (4) |  |  |  |

| Page Break |  |
| --- | --- |

| 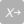 |
| --- |

Q30 In relation to improving sport performance, which of the following supplements do you think has NOT been supported by a strong body of scientific evidence? (Select only one)

- Caffeine (1)
- Ferulic Acid (2)
- Bicarbonate (3)
- Leucine (4)
- Not Sure (5)

| Page Break |  |
| --- | --- |

| 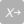 |
| --- |

Q31 Which of the following supplement do you think has been banned by NCAA when exceeding a specific urine threshold?
 (Select only one)

- Caffeine (1)
- Bicarbonate (2)
- Carnitine (3)
- Glycerol (4)
- Not Sure (5)

| Page Break |  |
| --- | --- |

| 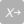 |
| --- |

Q32 Use of contaminated nutritional supplements can lead to a positive doping test. (Select only one)

- Yes (1)
- No (2)
- Not Sure (3)

| Page Break |  |
| --- | --- |

| 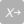 |
| --- |

Q33 Are you familiar with banned substances that may occur in nutritional supplements listed on the WADA (world anti-doping agency) or your own sport specific association? (Select only one)

- Yes (1)
- No (2)
- Not Sure (3)

| Page Break |  |
| --- | --- |

End of Block: Supplement Knowledge

Start of Block: Nutritional supplement use

Q36 **Nutritional Supplement Use**

| 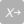 |
| --- |

Q38 Do you purchase or use nutritional supplements outside what is provided by your Athletic Department?
(Only select one)

- Yes (1)
- No (0)

| Page Break |  |
| --- | --- |

| 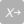 |
| --- |

Q39 **Please read the information below for answering the following question:**
Third-party testing is when an organization that is not the supplement company itself (hence, the third-party) evaluates a dietary supplement and vouches for its quality,” (United States Anti-Doping Agency, 2022). There are multiple third-party testing organizations that work to certify supplements. Athletes should preferably select these third-party tested products from organizations that test supplements for banned substances.

 What was the frequency of your purchasing third-party tested supplements during the last 12 months?
 (Only select one)

- Always (1)
- Most of the times (2)
- Sometimes (3)
- Never (4)

| Page Break |  |
| --- | --- |

| 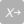 |
| --- |

Q48 I know where to find and order third-party tested supplements.
(Only select one)

- Agree (1)
- Disagree (0)
- Not Sure (2)

| Page Break |  |
| --- | --- |

|  |
| --- |

Q46 Check all boxes of third-party testing systems icons that you recognize from products that you have used during the last 12 months.
(Check all that apply)

- Image:Informed choice (1)
- Image:Informed sport (2)
- Image:Nsf certified for sport blue and orange (3)
- Image:Nsf international (4)
- Image:Usp (5)
- Image:Consumer lab logo (6)
- Image:Bscg logo (7)
- Image:Nzvt (8)
- Image:Kolner liste (9)
- I do not recognize any of these icons (10)

| Page Break |  |
| --- | --- |

| 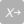 |
| --- |

Q40 If you use supplements or sports foods that your Athletic Department is not providing, who is purchasing them?
(Check all that apply)

- I am (1)
- Parents (2)
- Other (3)
- I never use nutritional supplements outside of what the Athletic Department offers me (4)

| Page Break |  |
| --- | --- |

| 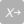 |
| --- |

Q41 Where do you purchase your nutritional supplements?
(Check all that apply)

- Brick and mortar store (1)
- Online/ecommerce (2)
- Directly from brand (self-paid) (3)
- Directly from brand (sponsored) (4)
- Indirect via my Athletic Department (self-paid) (5)
- Indirect via my Athletic Department (sponsored) (6)
- I do not purchase supplements (7)

| Page Break |  |
| --- | --- |

| 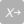 |
| --- |

Q43 Please check all of the nutritional supplements you have used during the last 12 months.
(Check all that apply, read carefully as they are in random order!)

- Multivitamin and mineral supplement (1)
- Combination of vitamins (2)
- Combination of minerals (3)
- Single vitamin (for example but not limited to vitamin C, vitamin D, and vitamin E) (4)
- Single mineral (for example but not limited to calcium, iron, magnesium, and zinc) (5)
- Fish oil/ essential fatty acids (6)
- Sports drink (for example but not limited to Powerade, and Gatorade) (7)
- Energy drink (for example but not limited to Red Bull, Monster and other energy drinks) (8)
- Energy gel or chewies (for example but not limited to Gu, Gatorade, and Powerbar) (9)
- Recovery drink (for example but not limited to Muscle Milk, and Rocking Refuel) (10)
- Protein shake (11)
- Weight gainer (12)
- Chocolate milk (or other flavored milk option with sugar added like strawberry milk) (13)
- Sports bar (for example but not limited to an energy bar, and protein bar) (14)
- Energy gel (15)
- Pre-workout supplement (16)
- BCAA (17)
- Leucine (18)
- Beta-Alanine (19)
- Dietary nitrate (for example but not limited to beetroot juice) (20)
- Caffeine (21)
- CLA (22)
- Creatine (23)
- Glucosamine (24)
- Herbs (such as: echinacea, ginseng, and ginkgo biloba or others herbs) (25)
- L-carnitine (26)
- Medium-chain triglycerides (MCT) (27)
- Sodium bicarbonate (28)
- Probiotics (29)
- Ribose (30)
- Quercitine (31)
- Tart Cherry (or other cherry varieties) (32)
- Exotic berries (for example but not limited to acai, and goji) (33)
- HMB (34)
- Ephedra (35)
- Tribulus terrestris (36)
- Maca root powder (37)
- Glycerol (38)
- Colostrum (39)
- CBD (Cannabidiol) (40)
- SARMs (such as: Ostarine, Andarine, Ligandrol (LGD-4033), and RAD140) (41)
- Fenugreek (or also known as: Methi) (42)
- Aswagandha (or also known as: Indian ginseng, poison gooseberry, winter cherry) (43)
- Dendrobium (44)
- Methylliberine (or also known as; dynamine, tetramethylurate or tetramethyluric acid) (45)
- Longjack (46)
- Kava (or also known as: kava kava, awa, ava, yaqona, yagona, seka, malok or malogu) (47)
- Phyllanthus (or leafflower) (48)
- Collagen (51)
- Glutamine (52)
- Co-enzyme Q10 (also known as: CoQ10) (53)
- Muscle cramp relievers (such as supplements based on: pickle juice, menthol, and sour, bitter or spicy substances) (54)
- Deer Antler Velvet extract (55)
- Other (49)
- None of the above (0)

| Page Break |  |
| --- | --- |

Display This Question:

If Please check all of the nutritional supplements you have used during the last 12 months. (Check a... = Other

Q44 Please write down any other supplements that you have used in the last 12 months that were not listed just above.

________________________________________________________________

| Page Break |  |
| --- | --- |

Display This Question:

If Please check all of the nutritional supplements you have used during the last 12 months. (Check a... != None of the above

Carry Forward Selected Choices from "Please check all of the nutritional supplements you have used during the last 12 months. (Check all that apply, read carefully as they are in random order!)"

| 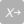 |
| --- |

Q107 Please score each supplement purchased or used during the last 12 months as to whether it was:

 -Third-party tested by an organization that tests for banned substances, such as NSF Certified for Sports, Informed Choice, Informed Sports, BSCG, or HASTA (**select: Certification with banned substance testing**).

 -Third-party certified by an organization that does not test for banned substances, such as the regular NSF and USP (**select: Certification without banned substance testing**).

 -In case you are not sure you can select not sure (**select: Not sure**).

|  | Certification with banned substance testing (1) | Certification without banned substance testing (2) | Not Sure (3) |
| --- | --- | --- | --- |
| Multivitamin and mineral supplement (x1) |  |  |  |
| Combination of vitamins (x4) |  |  |  |
| Combination of minerals (x5) |  |  |  |
| Single vitamin (for example but not limited to vitamin C, vitamin D, and vitamin E) (x6) |  |  |  |
| Single mineral (for example but not limited to calcium, iron, magnesium, and zinc) (x7) |  |  |  |
| Fish oil/ essential fatty acids (x8) |  |  |  |
| Sports drink (for example but not limited to Powerade, and Gatorade) (x3) |  |  |  |
| Energy drink (for example but not limited to Red Bull, Monster and other energy drinks) (x9) |  |  |  |
| Energy gel or chewies (for example but not limited to Gu, Gatorade, and Powerbar) (x10) |  |  |  |
| Recovery drink (for example but not limited to Muscle Milk, and Rocking Refuel) (x11) |  |  |  |
| Protein shake (x12) |  |  |  |
| Weight gainer (x13) |  |  |  |
| Chocolate milk (or other flavored milk option with sugar added like strawberry milk) (x14) |  |  |  |
| Sports bar (for example but not limited to an energy bar, and protein bar) (x15) |  |  |  |
| Energy gel (x16) |  |  |  |
| Pre-workout supplement (x2) |  |  |  |
| BCAA (x17) |  |  |  |
| Leucine (x18) |  |  |  |
| Beta-Alanine (x19) |  |  |  |
| Dietary nitrate (for example but not limited to beetroot juice) (x20) |  |  |  |
| Caffeine (x21) |  |  |  |
| CLA (x22) |  |  |  |
| Creatine (x23) |  |  |  |
| Glucosamine (x24) |  |  |  |
| Herbs (such as: echinacea, ginseng, and ginkgo biloba or others herbs) (x25) |  |  |  |
| L-carnitine (x26) |  |  |  |
| Medium-chain triglycerides (MCT) (x27) |  |  |  |
| Sodium bicarbonate (x28) |  |  |  |
| Probiotics (x29) |  |  |  |
| Ribose (x30) |  |  |  |
| Quercitine (x31) |  |  |  |
| Tart Cherry (or other cherry varieties) (x32) |  |  |  |
| Exotic berries (for example but not limited to acai, and goji) (x33) |  |  |  |
| HMB (x34) |  |  |  |
| Ephedra (x35) |  |  |  |
| Tribulus terrestris (x36) |  |  |  |
| Maca root powder (x37) |  |  |  |
| Glycerol (x38) |  |  |  |
| Colostrum (x39) |  |  |  |
| CBD (Cannabidiol) (x40) |  |  |  |
| SARMs (such as: Ostarine, Andarine, Ligandrol (LGD-4033), and RAD140) (x41) |  |  |  |
| Fenugreek (or also known as: Methi) (x42) |  |  |  |
| Aswagandha (or also known as: Indian ginseng, poison gooseberry, winter cherry) (x43) |  |  |  |
| Dendrobium (x44) |  |  |  |
| Methylliberine (or also known as; dynamine, tetramethylurate or tetramethyluric acid) (x45) |  |  |  |
| Longjack (x46) |  |  |  |
| Kava (or also known as: kava kava, awa, ava, yaqona, yagona, seka, malok or malogu) (x47) |  |  |  |
| Phyllanthus (or leafflower) (x48) |  |  |  |
| Collagen (x51) |  |  |  |
| Glutamine (x52) |  |  |  |
| Co-enzyme Q10 (also known as: CoQ10) (x53) |  |  |  |
| Muscle cramp relievers (such as supplements based on: pickle juice, menthol, and sour, bitter or spicy substances) (x54) |  |  |  |
| Deer Antler Velvet extract (x55) |  |  |  |
| Other (x49) |  |  |  |
| None of the above (x50) |  |  |  |

| Page Break |  |
| --- | --- |

Display This Question:

If Please check all of the nutritional supplements you have used during the last 12 months. (Check a... != None of the above

Carry Forward Selected Choices from "Please check all of the nutritional supplements you have used during the last 12 months. (Check all that apply, read carefully as they are in random order!)"

| 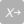 |
| --- |

Q108 How often do you use these supplements?
(Select one)

|  | At least once a day (1) | At least once a week (2) | At least once a month (3) | At least once a year (4) |
| --- | --- | --- | --- | --- |
| Multivitamin and mineral supplement (x1) |  |  |  |  |
| Combination of vitamins (x4) |  |  |  |  |
| Combination of minerals (x5) |  |  |  |  |
| Single vitamin (for example but not limited to vitamin C, vitamin D, and vitamin E) (x6) |  |  |  |  |
| Single mineral (for example but not limited to calcium, iron, magnesium, and zinc) (x7) |  |  |  |  |
| Fish oil/ essential fatty acids (x8) |  |  |  |  |
| Sports drink (for example but not limited to Powerade, and Gatorade) (x3) |  |  |  |  |
| Energy drink (for example but not limited to Red Bull, Monster and other energy drinks) (x9) |  |  |  |  |
| Energy gel or chewies (for example but not limited to Gu, Gatorade, and Powerbar) (x10) |  |  |  |  |
| Recovery drink (for example but not limited to Muscle Milk, and Rocking Refuel) (x11) |  |  |  |  |
| Protein shake (x12) |  |  |  |  |
| Weight gainer (x13) |  |  |  |  |
| Chocolate milk (or other flavored milk option with sugar added like strawberry milk) (x14) |  |  |  |  |
| Sports bar (for example but not limited to an energy bar, and protein bar) (x15) |  |  |  |  |
| Energy gel (x16) |  |  |  |  |
| Pre-workout supplement (x2) |  |  |  |  |
| BCAA (x17) |  |  |  |  |
| Leucine (x18) |  |  |  |  |
| Beta-Alanine (x19) |  |  |  |  |
| Dietary nitrate (for example but not limited to beetroot juice) (x20) |  |  |  |  |
| Caffeine (x21) |  |  |  |  |
| CLA (x22) |  |  |  |  |
| Creatine (x23) |  |  |  |  |
| Glucosamine (x24) |  |  |  |  |
| Herbs (such as: echinacea, ginseng, and ginkgo biloba or others herbs) (x25) |  |  |  |  |
| L-carnitine (x26) |  |  |  |  |
| Medium-chain triglycerides (MCT) (x27) |  |  |  |  |
| Sodium bicarbonate (x28) |  |  |  |  |
| Probiotics (x29) |  |  |  |  |
| Ribose (x30) |  |  |  |  |
| Quercitine (x31) |  |  |  |  |
| Tart Cherry (or other cherry varieties) (x32) |  |  |  |  |
| Exotic berries (for example but not limited to acai, and goji) (x33) |  |  |  |  |
| HMB (x34) |  |  |  |  |
| Ephedra (x35) |  |  |  |  |
| Tribulus terrestris (x36) |  |  |  |  |
| Maca root powder (x37) |  |  |  |  |
| Glycerol (x38) |  |  |  |  |
| Colostrum (x39) |  |  |  |  |
| CBD (Cannabidiol) (x40) |  |  |  |  |
| SARMs (such as: Ostarine, Andarine, Ligandrol (LGD-4033), and RAD140) (x41) |  |  |  |  |
| Fenugreek (or also known as: Methi) (x42) |  |  |  |  |
| Aswagandha (or also known as: Indian ginseng, poison gooseberry, winter cherry) (x43) |  |  |  |  |
| Dendrobium (x44) |  |  |  |  |
| Methylliberine (or also known as; dynamine, tetramethylurate or tetramethyluric acid) (x45) |  |  |  |  |
| Longjack (x46) |  |  |  |  |
| Kava (or also known as: kava kava, awa, ava, yaqona, yagona, seka, malok or malogu) (x47) |  |  |  |  |
| Phyllanthus (or leafflower) (x48) |  |  |  |  |
| Collagen (x51) |  |  |  |  |
| Glutamine (x52) |  |  |  |  |
| Co-enzyme Q10 (also known as: CoQ10) (x53) |  |  |  |  |
| Muscle cramp relievers (such as supplements based on: pickle juice, menthol, and sour, bitter or spicy substances) (x54) |  |  |  |  |
| Deer Antler Velvet extract (x55) |  |  |  |  |
| Other (x49) |  |  |  |  |
| None of the above (x50) |  |  |  |  |

End of Block: Nutritional supplement use

Start of Block: Attitude and barriers towards nutritional supplement use

Q47 **Attitude and barriers towards nutritional supplement use**

| 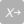 |
| --- |

Q103 I know where to find third-party tested supplements.
(Only select one)

- Agree (1)
- Disagree (0)
- Not Sure (2)

| Page Break |  |
| --- | --- |

| 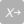 |
| --- |

Q104 I know where to order third-party tested supplements.
(Only select one)

- Agree (1)
- Disagree (0)
- Not Sure (2)

| Page Break |  |
| --- | --- |

| 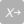 |
| --- |

Q57 I discuss all my supplement choices with the Athletic Departmental Sports RD.
(Only select one)

- Agree (1)
- Disagree (0)
- Not Sure (2)

| Page Break |  |
| --- | --- |

| 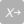 |
| --- |

Q58 If a teammate uses a supplement, I am more likely to try it as well.
(Only select one)

- Agree (1)
- Disagree (0)
- Not Sure (2)

| Page Break |  |
| --- | --- |

| 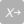 |
| --- |

Q61 The availability of supplements and sports foods within my Athletic Department reduces the need to purchase supplements on my own.
(Only select one)

- Agree (1)
- Somewhat Agree (2)
- Not Sure (3)
- Somewhat Disagree (4)
- Disagree (5)

| Page Break |  |
| --- | --- |

| 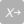 |
| --- |

Q105 The inaccessibility of certain supplements and sports foods within my Athletic Department, which I currently purchase on my own, increases my risk of testing positive.
(Only select one)

- Agree (1)
- Somewhat Agree (2)
- Not Sure (3)
- Somewhat Disagree (4)
- Disagree (5)

| Page Break |  |
| --- | --- |

| 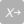 |
| --- |

Q62 I feel that my risk of testing positive would be reduced if I could purchase the supplements that I now purchase on my own via my Athletic Department.
(Only select one)

- Agree (1)
- Somewhat Agree (2)
- Not Sure (3)
- Somewhat Disagree (4)
- Disagree (5)

| Page Break |  |
| --- | --- |

| 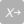 |
| --- |

Q63 If my Athletic Department could provide a range of evidence-based performance-enhancing supplements fitting my personal needs, then I would be willing not to purchase supplements externally.
 (Only select one)

- Agree (1)
- Somewhat Agree (2)
- Not Sure (3)
- Somewhat Disagree (4)
- Disagree (5)

| Page Break |  |
| --- | --- |

| 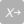 |
| --- |

Q65 I feel that the Sports RD and other staff within my Athletic Department send out inconsistent signals about the use of dietary supplements.
(Only select one)

- Agree (1)
- Somewhat Agree (2)
- Not Sure (3)
- Somewhat Disagree (4)
- Disagree (5)

| Page Break |  |
| --- | --- |

| 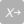 |
| --- |

Q66 I am uncomfortable discussing my supplement use with my Sports RD because I am afraid of a negative reaction, potentially resulting in the advice to stop using supplements I like to use.
(Only select one)

- Agree (1)
- Somewhat Agree (2)
- Not Sure (3)
- Somewhat Disagree (4)
- Disagree (5)

| Page Break |  |
| --- | --- |

| 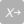 |
| --- |

Q71 I've decided to purchase one or more supplements as a result of the advice of family, friends, or teammates.
(Only select one)

- Agree (1)
- Disagree (0)
- Not Sure (2)

| Page Break |  |
| --- | --- |

| 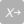 |
| --- |

Q80 What type of information about nutrition supplements and banned substances would you be interested in learning about?
(Check all that apply)

- How a product becomes contaminated (1)
- What types of products are usually most “risky” (2)
- Who certifies sport nutrition supplements that are safe to use (3)
- How to find tested products that are safe to use (4)
- How to purchase products that are safe to use (5)

End of Block: Attitude and barriers towards nutritional supplement use

Start of Block: Nutritional Supplements Section 1

Q1 The following questions will refer to your use of nutritional supplements. Nutritional supplements are for example: Dietary supplements: All vitamins, minerals, and essential fatty acids. Sport foods: Sports drinks (such as Gatorade, Powerade), energy drinks (such as Red Bull, Monster, Celsius), protein shakes, sports bars and energy gels. Performance supplements (or also called: ergogenic supplements): These are supplements that go with a performance-enhancing claim such as creatine, caffeine, pre-workout supplements and so on. Please answer the following questions as honestly as you can. There are no right or wrong answers. Please choose the answer you think is the most honest one for you.

| Page Break |  |
| --- | --- |

Q2 Using nutritional supplements to support my health and performance is:

|  | Unpleasant | Pleasant |
| --- | --- | --- |

|  | 1 | 2 | 3 | 4 | 5 | 6 | 7 |
| --- | --- | --- | --- | --- | --- | --- | --- |

| Tab on slider to activate score () | 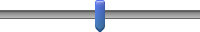 |
| --- | --- |

Q3 Using nutritional supplements to support my health and performance is:

|  | Harmful | Beneficial |
| --- | --- | --- |

|  | 1 | 2 | 3 | 4 | 5 | 6 | 7 |
| --- | --- | --- | --- | --- | --- | --- | --- |

| Tab on slider to activate score () | 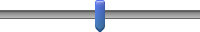 |
| --- | --- |

Q4 Using nutritional supplements to support my health and performance is:

|  | Useless | Useful |
| --- | --- | --- |

|  | 1 | 2 | 3 | 4 | 5 | 6 | 7 |
| --- | --- | --- | --- | --- | --- | --- | --- |

| Tab on slider to activate score () | 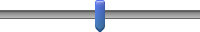 |
| --- | --- |

Q5 Using nutritional supplements to support my health and performance is:

|  | Unenjoyable | Enjoyable |
| --- | --- | --- |

|  | 1 | 2 | 3 | 4 | 5 | 6 | 7 |
| --- | --- | --- | --- | --- | --- | --- | --- |

| Tab on slider to activate score () | 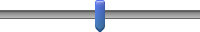 |
| --- | --- |

Q6 Using nutritional supplements to support my health and performance is:

|  | Unhealthy | Healthy |
| --- | --- | --- |

|  | 1 | 2 | 3 | 4 | 5 | 6 | 7 |
| --- | --- | --- | --- | --- | --- | --- | --- |

| Tab on slider to activate score () | 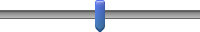 |
| --- | --- |

End of Block: Nutritional Supplements Section 1

Start of Block: Nutritional Supplements Section 2

Q1 My (head) sports coach believes that I should use nutritional supplements to support my health and performance.

|  | Strongly disagree | Strongly agree |
| --- | --- | --- |

|  | 1 | 2 | 3 | 4 | 5 | 6 | 7 |
| --- | --- | --- | --- | --- | --- | --- | --- |

| Tab on slider to activate score () | 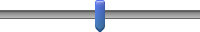 |
| --- | --- |

Q2 I am motivated to follow my head sports coach's beliefs that I should use nutritional supplements to support my health and performance.

|  | Not motivated | Very motivated |
| --- | --- | --- |

|  | 1 | 2 | 3 | 4 | 5 | 6 | 7 |
| --- | --- | --- | --- | --- | --- | --- | --- |

| Tab on slider to activate score () | 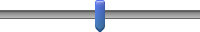 |
| --- | --- |

| Page Break |  |
| --- | --- |

Q3 My strength & conditioning coach believes that I should use nutritional supplements to support my health and performance.

|  | Strongly disagree | Strongly agree |
| --- | --- | --- |

|  | 1 | 2 | 3 | 4 | 5 | 6 | 7 |
| --- | --- | --- | --- | --- | --- | --- | --- |

| Tab on slider to activate score () | 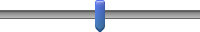 |
| --- | --- |

Q4 I am motivated to follow my strength & conditioning coach’s beliefs that I should use nutritional supplements to support my health and performance.

|  | Not motivated | Very motivated |
| --- | --- | --- |

|  | 1 | 2 | 3 | 4 | 5 | 6 | 7 |
| --- | --- | --- | --- | --- | --- | --- | --- |

| Tab on slider to activate score () | 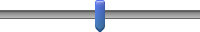 |
| --- | --- |

| Page Break |  |
| --- | --- |

Q5 My athletic trainer believes that I should use nutritional supplements to support my health and performance.

|  | Strongly disagree | Strongly agree |
| --- | --- | --- |

|  | 1 | 2 | 3 | 4 | 5 | 6 | 7 |
| --- | --- | --- | --- | --- | --- | --- | --- |

| Tab on slider to activate score () | 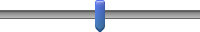 |
| --- | --- |

Q6 I am motivated to follow my athletic trainer’s beliefs that I should use nutritional supplements to support my health and performance.

|  | Not motivated | Very motivated |
| --- | --- | --- |

|  | 1 | 2 | 3 | 4 | 5 | 6 | 7 |
| --- | --- | --- | --- | --- | --- | --- | --- |

| Tab on slider to activate score () | 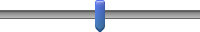 |
| --- | --- |

| Page Break |  |
| --- | --- |

Q7 My parents/guardians believe that I should use nutritional supplements to support my health and performance.

|  | Strongly disagree | Strongly agree |
| --- | --- | --- |

|  | 1 | 2 | 3 | 4 | 5 | 6 | 7 |
| --- | --- | --- | --- | --- | --- | --- | --- |

| Tab on slider to activate score () | 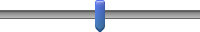 |
| --- | --- |

Q8 I am motivated to follow my parents/guardian’s beliefs that I should use nutritional supplements to support my health and performance.

|  | Not motivated | Very motivated |
| --- | --- | --- |

|  | 1 | 2 | 3 | 4 | 5 | 6 | 7 |
| --- | --- | --- | --- | --- | --- | --- | --- |

| Tab on slider to activate score () | 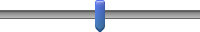 |
| --- | --- |

| Page Break |  |
| --- | --- |

Q9 My teammates believe that I should use nutritional supplements to support my health and performance.

|  | Strongly disagree | Strongly agree |
| --- | --- | --- |

|  | 1 | 2 | 3 | 4 | 5 | 6 | 7 |
| --- | --- | --- | --- | --- | --- | --- | --- |

| Tab on slider to activate score () | 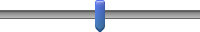 |
| --- | --- |

Q10 I am motivated to follow my teammates beliefs that I should use nutritional supplements to support my health and performance.

|  | Not motivated | Very motivated |
| --- | --- | --- |

|  | 1 | 2 | 3 | 4 | 5 | 6 | 7 |
| --- | --- | --- | --- | --- | --- | --- | --- |

| Tab on slider to activate score () | 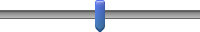 |
| --- | --- |

| Page Break |  |
| --- | --- |

Q134 My Sports RD (Registered Dietitian) believes that I should use nutritional supplements to support my health and performance.

|  | Strongly disagree | Strongly agree |
| --- | --- | --- |

|  | 1 | 2 | 3 | 4 | 5 | 6 | 7 |
| --- | --- | --- | --- | --- | --- | --- | --- |

| Tab on slider to activate score () | 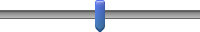 |
| --- | --- |

Q135 I am motivated to follow my Sports RD's beliefs that I should use nutritional supplements to support my health and performance.

|  | Not motivated | Very motivated |
| --- | --- | --- |

|  | 1 | 2 | 3 | 4 | 5 | 6 | 7 |
| --- | --- | --- | --- | --- | --- | --- | --- |

| Tab on slider to activate score () | 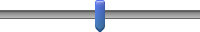 |
| --- | --- |

End of Block: Nutritional Supplements Section 2

Start of Block: Nutritional Supplements Section 3

Q1 It is under my control to use nutritional supplements to support my health and performance.

|  | Absolutely no control | Complete control |
| --- | --- | --- |

|  | 1 | 2 | 3 | 4 | 5 | 6 | 7 |
| --- | --- | --- | --- | --- | --- | --- | --- |

| Tab on slider to activate score () | 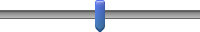 |
| --- | --- |

Q2 It is mostly up to me whether I use nutritional supplements to support my health and performance.

|  | Strongly disagree | Strongly agree |
| --- | --- | --- |

|  | 1 | 2 | 3 | 4 | 5 | 6 | 7 |
| --- | --- | --- | --- | --- | --- | --- | --- |

| Tab on slider to activate score () | 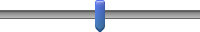 |
| --- | --- |

Q3 If I want to, I can use nutritional supplements to support my health and performance.

|  | Strongly disagree | Strongly agree |
| --- | --- | --- |

|  | 1 | 2 | 3 | 4 | 5 | 6 | 7 |
| --- | --- | --- | --- | --- | --- | --- | --- |

| Tab on slider to activate score () | 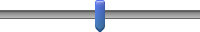 |
| --- | --- |

End of Block: Nutritional Supplements Section 3

Start of Block: Nutritional Supplements Section 4

Q1 I intend to use nutritional supplements to support my health and performance.

|  | Definitely do not | Definitely do |
| --- | --- | --- |

|  | 1 | 2 | 3 | 4 | 5 | 6 | 7 |
| --- | --- | --- | --- | --- | --- | --- | --- |

| Tab on slider to activate score () | 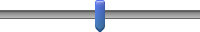 |
| --- | --- |

Q2 I am determined to use nutritional supplements to support my health and performance.

|  | Definitely false | Definitely true |
| --- | --- | --- |

|  | 1 | 2 | 3 | 4 | 5 | 6 | 7 |
| --- | --- | --- | --- | --- | --- | --- | --- |

| Tab on slider to activate score () | 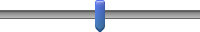 |
| --- | --- |

Q3 I plan to use nutritional supplements to support my health and performance.

|  | Definitely do not | Definitely do |
| --- | --- | --- |

|  | 1 | 2 | 3 | 4 | 5 | 6 | 7 |
| --- | --- | --- | --- | --- | --- | --- | --- |

| Tab on slider to activate score () | 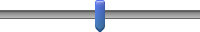 |
| --- | --- |

Q4 I have decided to use nutritional supplements to support my health and performance.

|  | Definitely false | Definitely true |
| --- | --- | --- |

|  | 1 | 2 | 3 | 4 | 5 | 6 | 7 |
| --- | --- | --- | --- | --- | --- | --- | --- |

| Tab on slider to activate score () | 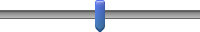 |
| --- | --- |

End of Block: Nutritional Supplements Section 4

Start of Block: End of Questionnaire Incentive

Q96 **Claim your incentive**

Q97 To ensure separate collection of your responses and your personal information you will be directed to another survey after finishing the current questionnaire.

To ensure we only reward athletes that filled out the full questionnaire we want to ask you to fill out a personal code below that you will only know. You will be asked to fill out this code again as part of the next questionnaire. Make sure to write it down before your finish this questionnaire as this code will allow to confirm your submission.

Q98 Please use random words (no names) and select a random number.
Please enter an eight (8) digit pin which includes four (4) letters and four (4) numbers

________________________________________________________________

Q99 After you click the forward button below you will be directed to a separate survey that will ask for your (8) digit pin, first and last name and your email address allowing the research team to provide you with your incentive.

End of Block: End of Questionnaire Incentive
